# Quality of life analysis in community pharmacy using deep learning and explainability methods

**DOI:** 10.1093/jamiaopen/ooag012

**Published:** 2026-01-30

**Authors:** María José Reyes-Medina, María Del Pilar Carrera-González, Vanesa Cantón-Habas, J L Ávila-Jiménez

**Affiliations:** Department of Nursing, Pharmacology and Physiotherapy, Faculty of Medicine and Nursing, University of Córdoba, Córdoba, 14004, Spain; Maimónides Institute of Biomedical Research of Córdoba (IMIBIC), Córdoba, 14004, Spain; Reina Sofia University Hospital, Córdoba, 14004, Spain; Department of Nursing, Pharmacology and Physiotherapy, Faculty of Medicine and Nursing, University of Córdoba, Córdoba, 14004, Spain; Maimónides Institute of Biomedical Research of Córdoba (IMIBIC), Córdoba, 14004, Spain; Department of Electronic and Computer Engineering, Andalusian Institute for Research in Data Science and Computational Intelligence (DaSCI), University of Córdoba, Córdoba, 14071, Spain

**Keywords:** chronic disease, pain, quality of life, community pharmacy services, deep learning, EQ-5D-5L

## Abstract

**Objectives:**

The study aimed to develop a deep learning-based model, using global and local explainability methods, to process clinical data collected in community pharmacies and identify the key variables influence health-related quality of life in patients with chronic diseases.

**Materials and Methods:**

Data from 347 chronic patients, including 257 variables, were analyzed. Five predictive models were compared using 10-way stratified cross-validation: Gradient Boosting, Random Forest, LightGBM, a fully connected neural network (FCNN), and a set of 5 FCNNs. For interpretability, SHapley Additive exPlanations (SHAP) was used for the global importance of variables and Local Interpretable Model-Agnostic Explanations (LIME) for the local interpretation of individual cases.

**Results:**

The FCNN ensemble achieved the best performance (*R*^2^ = 0.511 ± 0.126; 95% CI: 0.385-0.637; Mean Absolute Error = 0.0819 ± 0.0088; Mean Squared Error = 0.0122 ± 0.0039). Tree-based models showed slightly lower performance (eg, Gradient Boosting *R*^2^ = 0.484 ± 0.113). Explainability analysis identified pain, mobility limitations, beta-blocker use, anxiety/depression symptoms, and difficulties with activities of daily living as the most influential variables.

**Discussion:**

The findings highlight that deep learning models can capture complex relationships among multiple clinical and psychosocial variables. The combination of SHAP and LIME allows for clinically interpretable results, facilitating personalized decisions in chronic disease care. Furthermore, the accessibility of community pharmacies provides a practical setting for data collection and application of these predictive tools.

**Conclusions:**

The study demonstrates the potential of machine learning to support personalized decision-making in the management of chronic diseases from accessible settings such as community pharmacies, identifying the most important factors affecting patients’ quality of life.

## Introduction

The term quality of life, and in particular health-related quality of life (HRQoL), refers to the level of overall physical, mental, and social well-being derived from a person’s assessment of various domains of their life, considering the impact that their state of health has on these domains and not only referring to the absence of pathologies.^1^ The analysis of this variable provides a health outcome focused on how the patient feels, regardless of clinical data.^2^

HRQoL can be measured using an index that integrates several dimensions of the HRQoL construct.^3^ This multidimensional health indicator includes domains such as physical functioning, psychological well-being, emotional state, pain, and social functioning, which are affected by a person’s illness and/or treatment. It is a highly relevant tool for assessing the effect of diseases and the results of therapeutic interventions, with the aim of optimally planning and distributing healthcare resources and improving the patient’s quality of life.^4^

In this context, the population that is the target of intervention and the majority user of healthcare resources is the population with chronic conditions, mainly older adults, which is constantly growing. This is mainly due to increased life expectancy and, according to the latest demographic trends, this trend is expected to continue in the future. In fact, globally, it is predicted that by 2050, the population of people over 60 will double, reaching 77 years of age in optimal health conditions. This increase is due to factors such as improvements in social and health care, scientific advances, and a more active and healthy lifestyle.^5^

However, as longevity and/or life expectancy increase, although the functional and cognitive status of the individual deteriorates as a result of this life process, the probability of developing age-related diseases also increases, with the consequent onset of a state of prolonged dependence due to the individual’s disability resulting from the chronic nature of the process.^6^

In other words, although there has been a decrease in mortality among the older population, increased survival leads to an increase in chronic diseases. Most diseases, such as central nervous system disorders, diabetes, cancer, heart disease, stroke, as well as intestinal and kidney diseases, lead to an overall deterioration in well-being, limited performance and a reduction in quality of life.^7^

The ageing population is linked to an increase in the incidence of chronic diseases, which also carries with it a greater burden of pain in this population. It has thus been suggested that pain acts as a stress factor during ageing, accelerating functional deterioration and poor health. Various studies have shown that the prevalence of chronic pain in older people living in the community ranges from 40% to 75%.^8^^,^^9^ Chronic pain influences quality of life and can also lead to serious adverse outcomes, including disability and mortality.^10^^,^^11^ Increased pain is associated with greater deterioration in physical activities, muscle mass, muscle strength and autonomy and, therefore, a higher risk of developing sarcopenia.^12^^,^^13^ In addition, age-related sarcopenia is considered to be associated with chronic pain.^14^

Other common chronic conditions that are often linked to other non-cardiovascular diseases, like arthritis or chronic kidney disease, can cause a gradual decline and reduction in functional ability.^15^^,^^16^ In this complex clinical context, recent therapeutic guidelines have evolved to adapt to multimorbidity and optimize chronic treatment.^17^

In this regard, the European Society of Hypertension recently updated the category of beta-blockers, placing them on a par with thiazide diuretics, renin-angiotensin system antagonists (eg, angiotensin-converting enzyme inhibitors and angiotensin receptor antagonists) and calcium antagonists. This update reflects the broad clinical utility of beta-blockers, which are indicated for a variety of conditions that frequently coexisting with hypertension, such as ischemic heart disease, arrhythmias, and heart failure. Consequently, this change would allow the treatment of 2 diseases with a single drug (known as “dual action”). In most current national and international guidelines on hypertension, beta blockers are only considered an alternative when there are specific indications. Compared to other classes of first-line antihypertensive drugs, beta-blockers are significantly less effective in preventing stroke and cardiovascular mortality.^18^ In fact, according to the literature, beta-blockers may improve cardiovascular mortality, although the evidence is weak.^19^

All of this, combined with future epidemiological projections indicating that chronicity will continue to increase, will lead to intensive use of health services, especially in primary care.^20^

Other common conditions affecting the elderly population are depression and anxiety. These are common mental disorders that often coexist and are associated with increased disability and mortality.^21^ Thus, depression in older adults, in addition to being a significant disease in itself, is also associated with an increased risk of suicide.^22^^,^^23^ cardiovascular disease,^24–26^ certain types of cancer,^27^ and premature mortality.^28–30^ In this context, depressive disorders take on significant importance as they are one of the main causes of functional decline in old age.

Despite the availability of effective treatments, a significant proportion of depression cases in older adults remain undiagnosed, underscoring the importance of prevention and early identification.^31^^,^^32^ Research has highlighted a combination of behavioral and social factors that increase the risk of depression in later life, including excessive alcohol consumption, certain dietary patterns, loneliness, socioeconomic disadvantage, and bereavement.^33^^,^^34^ In addition to these psychosocial contributors, biological factors have also been implicated. For example, longitudinal evidence indicates that lower levels of insulin-like growth factor 1 (IGF-1) are associated with an increased risk of developing depressive symptoms in older adults, suggesting that endocrine pathways may play a role in the onset of late-life depression.^35^

According to recent literature, depression and anxiety have been identified as one of the main causes of disability in the older population. These disorders cannot only act as triggers, but also as risk factors or prodromal factors for the development of neurodegenerative diseases, such as dementia.^36^ Furthermore, depression and anxiety are, in themselves, predisposing factors for frailty in this age group, as they manifest through affective symptoms and are associated with significant cognitive impairment, medical comorbidities, and reduced functional capacity.^37^ In fact, recent studies support the hypothesis of an interdependent association between depressive symptoms and the use of antidepressant therapy, the presence of fear of falling, and the risk of falls.^38^

The combination of symptoms of depression and anxiety, such as feeling depressed or agitated, can contribute to impaired role functioning and interfere with self-care and mobility activities, even at milder levels of severity, when they may not even meet the threshold criteria for a clinical disorder.^39^^,^^40^ On the other hand, these symptoms can interact with each other when they occur together and become more severe and disabling over time.^41^^,^^42^

In this context, reducing the prevalence of chronic diseases and improving patients’ quality of life is a challenge not only for healthcare systems but also for society as a whole.^43^

However, the complexity of caring for chronic patients means that numerous groups must establish collaborations to provide a coordinated, efficient, and productive response to improve the quality of services offered to society.

Therefore, community pharmacies are particularly important, as they constitute the first level of healthcare and, as professionals who are close to the population, they focus their work on the demands of patients. They concentrate on aspects such as pharmacovigilance, the correct use of medicines, the promotion and prevention of non-communicable diseases, therapeutic adherence and the comprehensive management of chronicity and frailty.^44^ In fact, multidisciplinary programs that include pharmaceutical care reduce the risk of hospital visits and improve patients’ quality of life.^45^

In recent years, pharmaceutical services in community and hospital settings have seen significant advances. Among the most innovative developments is the creation of artificial intelligence (AI)-based applications and tools that help manage the treatment of high-risk patients.^46^

However, the development of AI-based tools that improve patient care requires in-depth prior study using deep learning models that enable us to obtain reliable models for predicting certain pathologies. Thus, the use of machine learning techniques, specifically deep learning, is showing promising results, as they efficiently process large amounts of clinical data and allow the creation of complex patient patterns that may sometimes go unnoticed by health experts.^47^

In short, the use of AI tools is characterized by greater accuracy, cost reduction and time optimization, while reducing potential human error.^48^

In conclusion, based on the above, the objective of this study is to create a machine learning model based on Deep Learning, using global [SHapley Additive exPlanations [SHAP]) and local (Local Interpretable Model-Agnostic Explanations [LIME]) explainability methods, capable of processing clinical data collected in Community Pharmacy, through the EQ-5D-5L questionnaire,^49^ which integrates several dimensions of the HRQoL construct, facilitating the identification of those variables that have a decisive impact on the individual’s quality of life. All this is done with the aim of enabling the precise identification of the most appropriate treatment, based on the sociodemographic and clinical characteristics of the patterns identified. This could contribute to reducing the frequency of new pathological diagnoses, thus improving the patient’s state of well-being.

## Methodology

### Description of the sample

The study sample consisted of people with chronic diseases, aged between 42 and 93 years (*M* = 67.04; SD = 10.41), who regularly visited community pharmacies in the town of Espejo (Córdoba, Spain) to collect their medication.

The inclusion criteria were: being 40 years of age or older, having at least one diagnosed chronic condition, and having signed the informed consent form to participate in the study.

Of the total number of participants, 59.4% (206) were women and 40.6% (141) were men. In terms of educational level, 48% (166) had completed primary education, 33% (115) had a lower educational level (no education or primary education), and 19% (66) had secondary or higher education.

### Dataset and variables

The data were sourced from internal records of a community pharmacy collected from January 2023 to June 2024 from 347 chronic patients.

The original dataset contains 258 (Table S1—see online supplementary material) variables, including responses to the EQ-5D-5L questionnaire, biochemical parameters from 58 serial laboratory tests, demographic information, and medication records.

### Data preprocessing

The preprocessing pipeline involved a series of systematic steps to ensure data quality and model readines**s.** Missing values were initially imputed using the median of each variable, preserving the central tendency and minimizing the impact of potential outliers. All continuous predictors were then standardized using *z*-score normalization to achieve zero mean and unit variance, facilitating the convergence and stability of subsequent models. To capture potential non-linearities and interactions among clinically relevant dimensions, feature engineering was applied. Specifically, interaction terms were introduced for *Mobility × Anxiety/Depression* and *Usual Activities × Anxiety/Depression*, along with a quadratic expansion of the *Pain/Discomfort* dimension (ie, [*Pain/Discomfort]*^2^). Lastly, the target variable, the EQ-5D Index, was also standardized to align with the modeling requirements and maintain numerical stability during training.

### Data splitting and validation

Model evaluation was conducted using stratified 10-fold cross-validation. This approach ensures homogeneous distribution of the EQ-5D Index across each fold by stratifying into quartiles, thus enhancing the robustness and generalization of the results.

### Predictive models evaluated

Four main predictive models were evaluated:

1. **Gradient Boosting:** A tree ensemble method optimized via boosting and evaluated using default parameters.^50^
2. **LightGBM:** A gradient boosting framework based on decision tree algorithms designed to be distributed and efficient.^51^
3. **Random Forest:** A classical ensemble of decision trees built with bootstrap sampling.^52^
4. **Fully Connected Neural Network (FCNN):** A deep learning model with a specific architecture, that comprises 4 hidden layers with 256-128-64-32 neurons, incorporating Batch Normalization, Dropout (*p* = 0.4), and a cyclic learning rate strategy.^53^

An ensemble strategy was also implemented for FCNNs. Five independently initialized FCNNs were trained using the same configuration, and their outputs were averaged to produce final predictions, thereby reducing model variance.

### Training strategy

**Figure 1.**
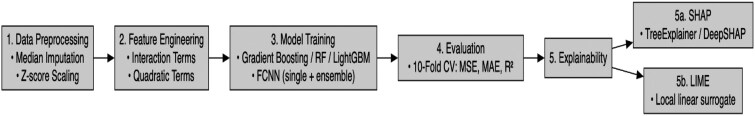
Representation of the machine learning pipeline used in this study. The workflow includes 5 main stages: (1) data preprocessing, comprising median imputation and standardization via *Z*-score scaling; (2) feature engineering, where interaction and quadratic terms were introduced to capture non-linear effects; (3) model training, involving tree-based models (Gradient Boosting, Random Forest, LightGBM) and FCNN, both individually and in ensemble; (4) model evaluation through stratified 10-fold cross-validation using MSE, MAE, and *R*^2^ metrics; and (5) model explainability, performed using SHAP (TreeExplainer or DeepSHAP) and LIME (local linear surrogate models).

Training of the neural networks was conducted in mini-batches of 128 samples, employing the AdamW optimizer with an initial learning rate of 1 e−4 and weight decay of 5 e−4. The learning rate was dynamically adjusted through a cyclic scheduler oscillating between 1 e−5 and 1 e−4. Additionally, early stopping was implemented, halting training if no significant improvement in validation loss occurred for 40 consecutive epochs. Numerical stability during training was enhanced by applying gradient clipping, limiting gradients to a maximum norm of 1.0.^54^ The complete flow of the methodological process is summarized in Figure 1.

### Evaluation metrics

Models were evaluated using Mean Squared Error (MSE) and Mean Absolute Error (MAE) to estimate absolute precision, as well as the coefficient of determination (*R*^2^), which assesses the overall model fit. Results were reported as mean ± SD across the 10 validation folds.

### Explainability analysis

To achieve a comprehensive and transparent interpretation of the results, 2 model explainability techniques were employed, SHAP and LIME.

SHapley Additive exPlanations,^55–57^ was used to estimate global variable importance, identifying features such as anxiety/depression, pain/discomfort, and mobility as the most influential. A SHAP value quantifies the contribution of each feature to a model’s prediction, drawing from cooperative game theory. Each feature is treated as a “player” in a coalition, and the model’s output is considered the “payout.” The SHAP value represents the average marginal contribution of a feature across all possible subsets of features.

**Figure 2.**
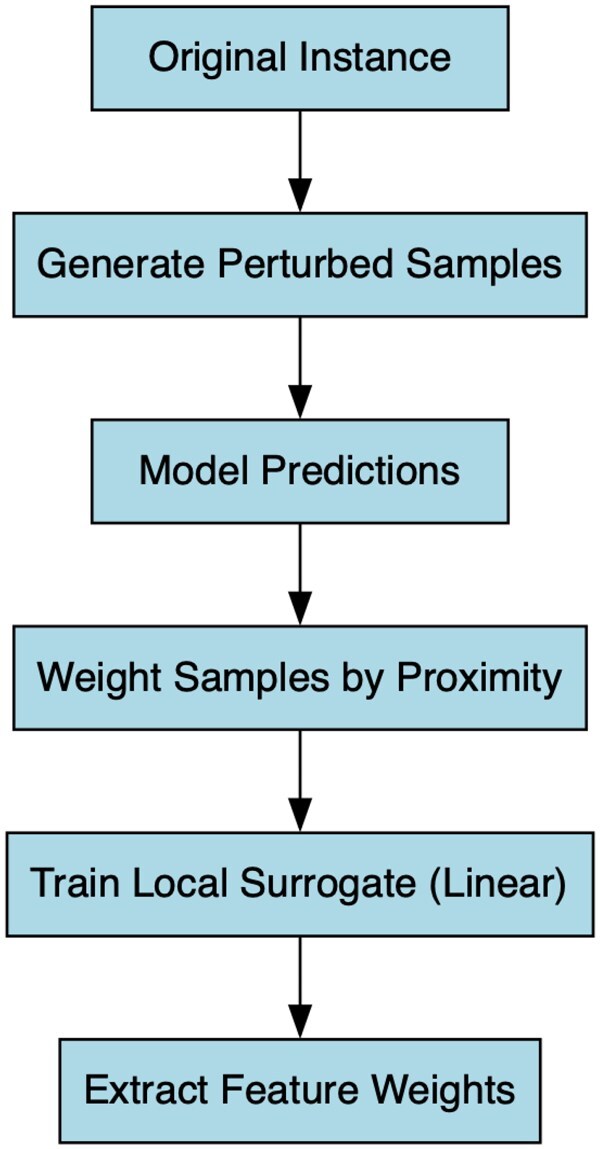
Workflow of the LIME algorithm. The method begins with the selection of an instance to explain, followed by the generation of perturbed samples around it. Predictions are then obtained from the original model. These samples are weighted based on their proximity to the original instance, and a linear model is trained locally. Finally, the feature weights are extracted to explain the prediction.

Local Interpretable Model-Agnostic Explanations,^58^ was applied to generate interpretable explanations of individual predictions, highlighting how specific variable combinations influenced output values. LIME generates a local linear surrogate model to explain individual predictions of complex “black-box” algorithms. Given a target instance, LIME creates numerous perturbed samples around it and obtains their predictions from the original model. These samples are weighted by proximity to the original instance, emphasizing local fidelity. A simple, interpretable model, typically a sparse linear regression, is trained on these weighted samples to approximate the black-box model’s behavior in that neighborhood. The resulting feature coefficients indicate the magnitude and direction of each variable’s local influence on the prediction, thereby providing an understandable explanation of why the model produced that specific output for the instance, as illustrated in Figure 2.

## Results

This section presents the predictive performance of the evaluated models and the corresponding explainability analyses. The results are based on a stratified 10-fold cross-validation strategy.

### Predictive performance

Table 1 summarizes the average predictive performance of each model over the 10 folds, reported as mean ± SD for MSE, MAE, and coefficient of determination (*R*^2^).

**Table 1.** Predictive performance (mean ± SD)

Model	MSE (↓)	MAE (↓)	*R* ^2^ (↑)
Decision Tree	0.0412 ± 0.0187	0.1156 ± 0.0199	0.236 ± 0.179
Random Forest	0.0243 ± 0.0116	0.1015 ± 0.0160	0.435 ± 0.152
Gradient Boosting	0.0212 ± 0.0119	0.0931 ± 0.0165	0.484 ± 0.158
LightGBM	0.0206 ± 0.0107	0.0943 ± 0.0143	0.496 ± 0.135
FCNN (single)	0.0185 ± 0.0107	0.0947 ± 0.0184	0.404 ± 0.211
FCNN (Ensemble) (x5)	**0.0122 ± 0.0054**	**0.0819 ± 0.0123**	**0.511 ± 0.176**

Values in bold indicate the best performance for each metric (lowest for MSE and MAE; highest for *R*²).

The ensemble FCNN model achieved the best overall results, outperforming both tree-based and single neural models in terms of all 3 evaluation metrics. The gain in *R*^2^ indicates enhanced generalization and reliability.

### Explainability analysis

#### SHAP value analysis

SHapley Additive exPlanations analysis was conducted to assess global feature importance using a surrogate model trained on the ensemble FCNN outputs. The 5 most influential features in the FCNN ensemble model were:

1. Pain Level (Item 4 EQ-5D-5L)
2. Mobility (Item 1 EQ-5D-5L)
3. Treatment with beta blockers
4. Anxiety/Depression (Item 5 EQ-5D-5L)
5. Usual Activities Difficulty (Item 3 EQ-5D-5L)

These variables displayed consistent SHAP impact across all folds and patients, reinforcing their clinical relevance.

#### LIME interpretation examples

To complement the global SHAP analysis, LIME was applied to interpret specific individual predictions from the FCNN ensemble with 2 selected representative examples (Table 2).

**Table 2.** Top contributing features identified by LIME for 2 representative instances.

Feature/Condition	LIME value (Instance 10)	LIME value (Instance 53)	Interpretation
Pain/Discomfort (Item 4, EQ-5D-5L)	+0.73	+0.73	Strong positive contribution to the predicted outcome.
Mobility (Item 1, EQ-5D-5L)	−0.71	−0.71	Large negative impact on the prediction.
Anxiety/Depression (Item 5, EQ-5D-5L)	−0.62	−0.62	Strong negative contribution, slightly lower than mobility.
Usual Activities (Item 3, EQ-5D-5L)	−0.29	−0.29	Moderate negative effect.
Self-care (Item 2, EQ-5D-5L)		−0.08	Minor negative contribution.
Selective serotonin reuptake inhibitors (SSRIs)		−0.15	Noticeable negative impact.
GPIIb/IIIa receptor blockers, vaginal atrophy, salicylates, tachycardia	−0.08 to −0.15		Moderate negative contributions.
Raynaud’s syndrome, metabolic syndrome, treatment with salicylates and arylacetics		−0.08 to −0.09	Soft but cumulative negative contributions.
Combined statin + calcium channel blocker therapy, leukemia, pancreatitis, etc.	−0.05		Slight negative influence.
Diabetic foot, treatment with antacids and meglitinins		−0.05	Minimal negative impact.

These cases showed on Table 2 illustrate the ability of LIME to highlight the local effect of specific variables on the model output. LIME explanations were found to be coherent with SHAP values and consistent with clinical expectations.

Taken together, SHAP and LIME provide both global and local explainability, enhancing the interpretability and trustworthiness of the FCNN ensemble’s predictions.

## Discussion

The use of machine learning models, particularly deep learning, has proven to be an innovative and effective tool for predicting quality of life in various clinical settings. These models allow for the analysis of large volumes of heterogeneous clinical data, including demographic variables, analytical data, and questionnaire responses, capturing complex and non-linear relationships that are beyond the scope of conventional statistical methods. The incorporation of global and local explainability techniques, such as SHAP and LIME, provides transparency and insight into the factors that most influence predictions, facilitating the clinical translation of results and guiding specific interventions.^59^^,^^60^

This integrated approach would not only improve clinical monitoring and patient-centerd care, but would also contribute to the efficiency and sustainability of health systems, especially in the management of chronic conditions that require a multidimensional and coordinated approach. In essence, data analysis and information management have enabled Deep Learning models to create algorithms for complex situations related to chronicity.^61^

In this study, specific Deep Learning models have been developed based on data from medical records and questionnaires to identify patterns indicative of health deterioration in terms of quality of life in chronic patients.

The results obtained have shown that variables such as pain, mobility, treatment with beta-blockers, and depression and/or anxiety have a decisive impact on the patient’s quality of life.^62^^,^^63^ To a more moderate extent, activities of daily living are also affected.

As mentioned above, pain leads to functional impairment, such as reduced mobility and limitations in activities of daily living, which in turn leads to difficulties in social participation and greater social isolation, factors that negatively affect psychological and social well-being. In fact, mobility difficulties represent a critical clinical manifestation that limits functionality and personal autonomy. Functional limitation is correlated with increased vulnerability to comorbidities and progressive deterioration, constituting an essential determinant in the overall assessment of health status.^64^ Therefore, chronic pain and emotional and functional status are prominent indicators of health status and disability worldwide.^65^ According to the scientific literature, interventions aimed at treating pain,^66^ loss of movement,^67^ and anxiety or depression,^68^ will have the greatest tangible impact on improving perceived health.

In this regard, our study points to the extremely significant impact of this variable on patients’ quality of life, as well as the variable of mobility, which is directly affected by the former. As we have seen, this information can be gathered by community pharmacists and validated by our model, with the consequent positive effect on patients’ quality of life and possibly reduced use of health services.

On the other hand, it is essential to consider how pharmacological treatment affects a patient’s quality of life, beyond its direct clinical effects. In this context, the use of beta-blockers emerged as one of the variables with the greatest predictive value in the model, suggesting a relevant association with the perception of health status in patients with chronic diseases. However, the available evidence regarding their impact on quality of life is ambiguous. For example, Martin et al. evaluated the effect of these drugs in patients with chronic heart failure with preserved ejection fraction, and although they observed a reduction in cardiovascular mortality, they did not find significant improvements in quality of life.^19^ In contrast, Steinman et al. analyzed the use of beta-blockers after a myocardial infarction, and concluded that, although they were associated with lower mortality, they also increased the risk of functional deterioration by ∼14%, which had a negative impact on the patients’ quality of life.^69^

In summary, while beta-blockers may reduce cardiovascular mortality in some patients, they do not appear to improve quality of life and may even be associated with functional decline in a significant percentage of patients after a heart attack.

Similarly, anxiety and depression are highly prevalent disorders in older adults and in patients with chronic diseases, and their prevalence is underdiagnosed in healthcare settings.^70^^,^^71^ Both conditions affect not only the interpretation of physical symptoms, but also the patient’s ability to cope with them, manage their treatment and maintain self-care behaviors. Mental health acts as a key modulator of overall health perception, influencing both the assessment of physical condition and the motivation to participate in daily and social activities. In addition to the patient’s objective situation in terms of comorbidity, these psychiatric conditions increase the subjective perception of disability, amplify suffering and impair resilience, resulting in a significant reduction in self-perceived quality of life.^72^

Depression and anxiety are pathologies linked to chronic pain and are not only clinically simultaneous, but also directly proportional. In this sense, we must not overlook the importance of changes in mood, as it is a predictor of daily functioning and, consequently, of body movement.^73^^,^^74^ In fact, from a psychosocial perspective, persistent pain creates a vicious cycle involving physical deterioration, social isolation, and emotional distress, contributing significantly to a decline in the perception of well-being and consequently negatively affects HRQoL, in addition to being incapacitating for the patient.^75^^,^^76^

In other words, the main factors affecting patients’ quality of life are the clinical consequences of chronic conditions that are common in the elderly population, such as pain, depression and/or anxiety, and mobility.

Using the EQ-5D-5L questionnaire, community pharmacists can identify at-risk patients early and, with validation from our model, prioritize interventions such as medication reviews or referrals to specialized services. Explainable techniques like SHAP and LIME allow these interventions to be tailored to the most relevant factors for each patient. This information facilitates multidisciplinary coordination within the healthcare team, improving functional autonomy, perception of well-being, and potentially reducing healthcare utilization. Thus, the model’s results reinforce the EQ-5D-5L data and offer a practical framework for evidence-based community interventions, supporting a multidisciplinary approach for patients with chronic conditions.^77^

## Conclusions

The implementation/development of a machine learning model, developed based on variables obtained in the context of community pharmacy, represents a remarkable opportunity to deepen our understanding of how different clinical factors (pain, mobility impairment, and anxiety/depression) impact patients’ perception of health, allowing for the development of follow-up and/or interventions in this regard. The machine learning model developed in our study reinforces the need for comprehensive approaches that prioritize interventions aimed at pain relief, adequate mental health management, and the promotion of physical functionality to optimize quality of life in populations with chronic conditions.

## Acknowledgment

Funding for open access charge: Universidad de Córdoba/CBUA.

## Supplementary Material

ooag012_Supplementary_Data

## Data Availability

The datasets generated and/or analyzed during the current study are not publicly available in order to ensure the privacy and confidentiality of study participants. Access to these data can be provided by the corresponding author upon reasonable request, subject to applicable ethical and legal restrictions.
