# Supplementary material for: Quality of life analysis in community pharmacy using deep learning and explainability methods
Source: JAMIA Open. 2026 Jan 30;9(1):ooag012. doi: 10.1093/jamiaopen/ooag012 (PMC12863085; doi:10.1093/jamiaopen/ooag012)
Supplement: ooag012_Supplementary_Data [file ooag012_supplementary_data.zip › Graphic summary.pdf]

Quality of Life Analysis in Community Pharmacy Using Deep Learning and Explainability Methods

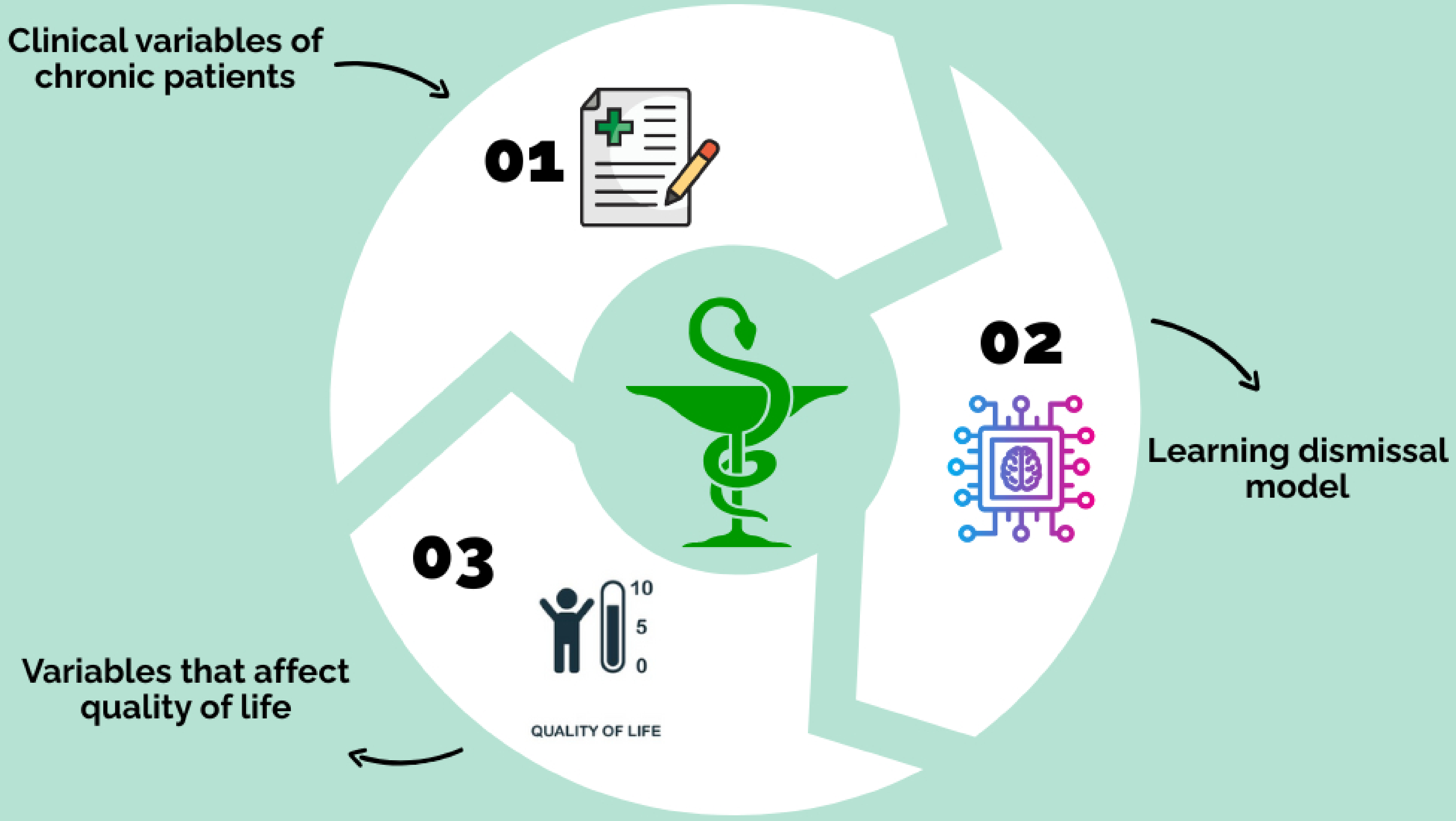

Monitoring clinical variables of chronic patients + learning dependency models = improved management of chronic patients
