# Supplementary material for: Quality of life analysis in community pharmacy using deep learning and explainability methods
Source: JAMIA Open. 2026 Jan 30;9(1):ooag012. doi: 10.1093/jamiaopen/ooag012 (PMC12863085; doi:10.1093/jamiaopen/ooag012)
Supplement: ooag012_Supplementary_Data [file ooag012_supplementary_data.zip › Supplementary table 1.docx]

**Supplementary Table 1. Variables used in the construction of the predictive model**

| **Variables extracted in community pharmacy** | | | |
| --- | --- | --- | --- |
| Age | Rhizarthrosis | Basal cell carcinoma | Indoleacetic acid derivatives |
| Sex | Gonarthrosis | Thrombocythemia | COX-2 selective inhibitors |
| Marital status | Depression | Colorectal cancer | NSAID (Non-Steroidal Anti-Inflammatory Drugs)+ gastroprotection combinations |
| Employment status | Angina pectoris | Other diseases (204) | Non-antiinflammatory antirheumatics |
| No. of pregnancies | Diabetic retinopathy | Elevated glucose levels | Muscle relaxants and anticonvulsants |
| No. of children | Osteopenia/Osteoporosis | Elevated HbA1c values | Other musculoskeletal system preparations |
| Dependent family member (No/Yes) | Glaucoma | Antihypertensives | Opioid analgesics |
| Educational level | Asthma | ACE inhibitors | Pyrazolones |
| Health care coverage | Vitamin B7 deficiency | ARBs (Angiotensin II receptor blockers) | Analgesic combinations with codeine and/or caffeine |
| Public | Extrasystoles | Diuretics | Antivertiginous agents |
| Private | Arrhythmia | ACE inhibitors + HCTZ | Antiepileptics used as analgesics |
| Tobacco consumption | Obesity | ARBs + HCTZ | Tramadol + other analgesic combination (paracetamol or dexketoprofen) |
| Alcohol consumption | Tachycardia | Beta-blockers | Oral corticosteroids |
| Stress level | Carpal tunnel syndrome | Beta-blockers + HCTZ | Antidepressants used as analgesics |
| Elevated BP values in Visit 1 | Chronic headache/migraine | Calcium channel blockers | Anxiolytics |
| Medical appointment | Vitamin D deficiency | ACEI/ARB + calcium channel blocker combination | Antidepressants |
| Drug withdrawal | Atherosclerosis | Calcium channel blocker + statin combination | Neuroleptics |
| Patient prepares own medication | Gastritis | Other antihypertensive drugs | Anticonvulsant/antiepileptic treatment |
| Treatment adherence | Solitary pulmonary nodule | Antiplatelet agents | Antiparkinson drugs |
| Pharmaceutical satisfaction | Colon adenoma | Anticoagulants | Intermediate/long-acting benzodiazepines |
| Mobility | IBS/Ulcerative colitis | Antiplatelet + anticoagulant combination | Short/intermediate-acting benzodiazepines |
| Self-care | Type 1 diabetes | Vasopressors | Other anxiolytics or hypnotics |
| Usual activities | Metabolic syndrome | Drugs acting on platelet activity mediators | Tricyclic antidepressants |
| Pain/discomfort | Gastric/duodenal ulcer | GP IIb/IIIa receptor blockers | Heterocyclic antidepressants |
| Anxiety/depression | Neck pain/cervicobrachialgia | ADP receptor antagonists | SSRIs (Selective Serotonin Reuptake Inhibitors) |
| Health status index (IS) | Diverticula | Coumarins | SNRIs (Serotonin–Norepinephrine Reuptake Inhibitors) |
| EQ-5D Index | Arterial lesion | Direct thrombin inhibitors | Short-acting β-adrenergic bronchodilators |
| 100-IS | Vulvar lichen sclerosus | Direct FXa inhibitors | Long-acting β-adrenergic bronchodilators |
| VAS (Visual Analogue Scale) | Non-alcoholic fatty liver disease | Indirect FXa inhibitors | LABA + inhaled corticosteroids |
| Sleeps well? | Chronic rhinitis | LMWH (Low Molecular Weight Heparin) | Short-acting anticholinergic bronchodilators |
| Epworth Sleepiness Scale | Postmenopausal vaginal dryness | Capillary stabilizer | Long-acting anticholinergic bronchodilators |
| Tingling in hands or feet | Type 2 diabetes | Other circulation drugs | LABA + LAMA (dual bronchodilator therapy) |
| Tingling in hands | Back pain/low back pain | Lipid-lowering agents | LABA + LAMA + ICS (triple therapy) |
| Tingling in feet | Dermatitis | Statins | Xanthines (bronchodilators) |
| Swollen feet | Psoriasis | Fibrates | Inhaled corticosteroids |
| Tinnitus | Pneumonia (lung infection) | Ion exchange resins | Leukotriene receptor antagonists |
| Hypertension | COPD | Cholesterol absorption inhibitors | Antihistamines |
| Prostate cancer | Ménière’s syndrome/vertigo | Monoclonal antibodies | Antihistamines + corticosteroids |
| Ventral hernia | Urinary incontinence | Statins + cholesterol absorption inhibitor | Mucolytics |
| Thyroid nodule | Carpal tunnel syndrome | Other dyslipidemia drugs | Oral corticosteroids |
| Lung cancer | Acute myocardial infarction | Oral antidiabetic drugs | Oxygen therapy |
| Neurological disorder, memory impairment | Kidney transplant | Insulin | Thyroid treatment |
| Gastroesophageal reflux (GERD) | Chronic kidney disease (CKD) | Biguanides | Vitamin D |
| Hyperuricemia | Other types of cancer | Sulfonylureas | Folic acid |
| Anxiety | Myoma | α-Glucosidase inhibitors | Iron |
| Insomnia | Alcoholic liver disease | Meglitinides | Calcium |
| Gout | Pancreatitis | Thiazolidinediones | Vitamin B12 |
| Hiatal hernia | Chronic leukemia | DPP-4 inhibitors | Other vitamins or minerals |
| Dyslipidemia | Diabetic foot | GLP-1 analogues | PPIs |
| Raynaud’s syndrome | Colon polyps | SGLT inhibitors | H2 receptor antagonists |
| Knee pain | Macular degeneration | Oral antidiabetic combinations | Mucosal protectants |
| Arm/shoulder pain | Varicose veins | Osteoporosis treatment | Antacids |
| Elbow pain | Hiatal insufficiency | Neuropathic pain | Prokinetics |
| Lives with pain | Gastric polyps | Salicylates | Other gastrointestinal drugs |
| Hypothyroidism | Uterine cancer | NSAIDs (Non-Steroidal Anti-Inflammatory Drugs) | Omeprazole or esomeprazole |
| Hyperthyroidism | Chronic pharyngitis | Muscle relaxants | Omeprazole |
| Fibromyalgia | Polyarthralgia | Antimigraine drugs | Esomeprazole |
| Dyspepsia/ulcer dyspepsia | Breast cancer | Antigout drugs | Antibiotics |
| Tremors | Cerebrovascular disease | Analgesics + antipyretics | Antifungals |
| Heart disease | Alcoholic toxic encephalopathy | Propionic acid derivatives | Prostate treatment |
| Benign prostatic hyperplasia (BPH) | Gilbert’s syndrome | Pyrroleacetic acid derivatives | Urinary incontinence treatment |
| Musculoskeletal disorder diagnosis | Chronic urticaria | Phenylacetic (arylacetic) derivatives | Immunosuppressive treatment |
| Rheumatoid arthritis | Pulmonary thromboembolism | Oxicams | Indoleacetic acid derivatives |
| Osteoarthritis | Encephalitis | Nicotinic acid derivatives |  |
| Herniated disc | Frontal meningioma | Anticonvulsants |  |
|  | | | |

ACE: Angiotensin-Converting Enzyme; HCTZ: Hydrochlorothiazide; ARB: Angiotensin II Receptor Blocker; VAS: Visual Analogue Scale; IS: Health Status Index; LABA: Long-Acting Beta-Agonist; LAMA: Long-Acting Muscarinic Antagonist; ICS: Inhaled Corticosteroid; COPD: Chronic Obstructive Pulmonary Disease; GLP-1: Glucagon-Like Peptide-1; DPP-4: Dipeptidyl Peptidase-4; SGLT: Sodium-Glucose Cotransporter; PPIs: Proton Pump Inhibitors
